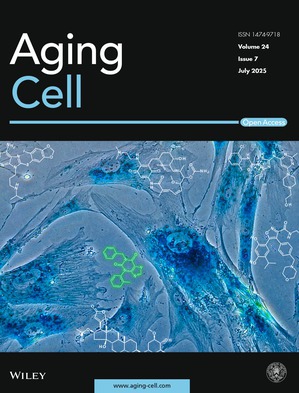# Additional Cover

**DOI:** 10.1111/acel.70171

**Published:** 2025-07-16

**Authors:** Sandra Atlante, Luca Cis, Davide Pirolli, Michela Gottardi Zamperla, Veronica Barbi, Antonello Mai, Clemens Zwergel, Serena Marcozzi, Maria Elisa Giuliani, Giorgia Bigossi, Giovanni Lai, Fiorenza Orlando, Robertina Giacconi, Fabrizia Lattanzio, Giulia Matacchione, Chiara Giordani, Massimo Bracci, Fabiola Olivieri, Federico Boschi, Paola Tabarelli De Fatis, Giovanni Battista Ivaldi, Marco Malavolta, Antonella Farsetti, Maria Cristina De Rosa, Carlo Gaetano

## Abstract

Cover legend: The cover image is based on the article *A Xanthine Derivative With Novel Heat Shock Protein 90‐Alpha Inhibitory and Senolytic Properties* by Sandra Atlante et al., https://doi.org/10.1111/acel.70047.